# Cost-effectiveness analysis of preventive strategies for child anxiety and depression: a health service perspective

**DOI:** 10.1186/s13034-025-00962-w

**Published:** 2025-10-01

**Authors:** Kristin D. Martinsen, Eline Aas, Jo Magne Ingul, Carina Lisøy, Frode Adolfsen, Lene-Mari Rasmussen, Tore Wentzel-Larsen, Simon-Peter Neumer

**Affiliations:** 1 Department of Psychology, Faculty of Social Sciences, University of Oslo, Forskningsveien 3A, 0373 Oslo, Norway; 2 Department of Health Management and Health Economics, The Medical Faculty, University of Oslo, Oslo, Norway; 3 Regional Centre for Child and Adolescent Mental Health, Eastern and Southern Norway, Oslo, Norway; 4 Faculty of Health Sciences Regional Centre for Child and Youth Mental Health and Child Welfare NorthUiT, The Arctic University of Norway, Tromsø, Norway; 5 Department of Mental Health, Faculty of Medicine and Health Sciences, Regional Centre for Child and Youth Mental Health and Child Welfare (RKBU), NTNU - Norwegian University of Science and Technology, Trondheim, Norway

**Keywords:** Cost effectiveness, Anxiety, Depression, Children, Prevention, Early intervention

## Abstract

**Background:**

The rising prevalence of mental health problems presents economic and social challenges with youth anxiety and depression as major contributors. While strengthening preventive measures is essential to mitigate the impacts of these conditions, limited resources necessitate careful prioritization of interventions. This underscores the need for cost-effectiveness studies to inform resource allocation and decision-making.

**Methods:**

This study utilized data from a trial employing the MOST framework to optimize a targeted group CBT intervention for youths exhibiting anxiety and depressive symptoms. Experimental strategies were evaluated for cost-effectiveness using real-world data examining intervention costs, child mental health outcomes: anxious and depressive symptoms and quality-adjusted life-years (QALYs). Three intervention factors were examined: delivery format (16 sessions vs. 8 sessions + 8 web-based sessions), parental involvement (five sessions vs. parental brochure), and measurement feedback system (MFS) (feedback vs. no feedback), producing eight intervention strategies. The study included 701 children aged 8 to 12, recruited from 52 public schools in 39 municipalities in Norway. Statistical analysis was conducted using R.

**Results:**

For depressive symptoms and QALYs, cost-effective strategies included the long in-person version with low parental involvement and no feedback, and the hybrid format (in-person + web-based sessions) with low parental involvement and no feedback. For depressive symptoms, the hybrid format with parental involvement and no feedback was a feasible strategy. For anxiety symptoms, cost-effective strategies involved the long in-person version with low parental involvement and feedback, and the hybrid format with low parental involvement and feedback.

**Discussion:**

In resource-constrained environments, the least resource-intensive strategies can ensure symptom reduction at minimal cost. Parental involvement is a viable alternative under conditions of intermediate resources, balancing costs, and clinical benefits. When symptom reduction is prioritized, and cost is secondary, the long in-person format with low parental involvement and no feedback may be preferable.

**Conclusion:**

The study highlights trade-offs between cost containment, reach, and intervention effectiveness. Policymakers must weigh costs against desired levels of symptom reduction when making decisions.

*Trial registration number* Clinical Trials NCT04263558 (Feb 11, 20, Jan 25. 21).

## Introduction

Given constraints in healthcare resources cost-effective solutions to address youth mental health challenges are urgently needed. Researchers have established the effectiveness of mental health interventions, and experts recognize prevention as a cornerstone in contemporary mental health strategy (e.g [1]). The cost-effectiveness of these preventive interventions however remains under-explored. This study investigates the economic sustainability of 8 different preventive delivery methods for childhood anxiety and depression when delivering the Emotion intervention [2]. The cost-effectiveness analysis aims to inform decision makers in refining resource allocation between different delivery strategies of the intervention.

An increasing number of people struggle with mental health problems which are a leading cause of disease burden [3] and incur significant economic costs globally [4]. In Norway, annual mental health costs are approximately NOK 340 billion [5], primarily due to treatment (12%) and indirect costs related to loss of education, and workforce participation. Notably, common conditions including anxiety and depression, account for the highest costs (FHI, 2016) and significantly reduce quality of life (e.g., [6]). Anxiety and depression often emerge early, and impair social and academic functioning and quality of life [7–9]. This underscores the critical importance of treating and preventing common mental health problems in youth. Mental health services have traditionally been treatment-oriented [10, 11], but there is a growing need for preventive efforts to address the rising prevalence and impact of mental health problems [12]. Limited resources make it challenging to choose interventions, highlighting the need for cost-effectiveness studies to guide resource allocation and inform decision-making.

Meta-analyses confirm the effectiveness of interventions targeting symptoms of anxiety and depression [13], although intervention costs vary. For instance, Lee, Barendregt [14] found that school-based internet-delivered prevention interventions were cost-effective compared to face-to face interventions. Additionally, Simon, Dirksen [15] identified the economic benefits of parent-focused interventions for children of anxious parents, and child-focused interventions for children of non-anxious parents. Despite research demonstrating their effectiveness, communities often do not adopt interventions [16, 17]. Tailoring interventions to their delivery context while aiming for effectiveness with minimal effort, is therefore essential [18]. Demonstrating the effectiveness, resource use, and cost-effectiveness of new interventions can drive the adoption and accessibility of effective interventions [19, 20].

The current study uses data from The Echo-study [21] which used The Multiphase Optimization Strategy (MOST, 19) to optimize and evaluate interventions. The main goal of the Echo-study was to test an optimized indicated group cognitive behavioral (CBT) intervention for youth with anxious and/or depressive symptoms (see 23). A secondary goal was to investigate which combination of factors give the best tradeoff between effect, usability, and cost-effectiveness. The present study uses an innovative approach where we evaluate the eight experimental strategies regarding cost-effectiveness using real-world data. We assess three key factors for optimizing child mental health: delivery format (16 group sessions vs. 8 group sessions + 8 web-based sessions), parental involvement (five group sessions vs. parental brochure), and a measurement feedback system (MFS) (feedback vs. no feedback) yielding eight strategies.

The study investigates health service costs associated with different delivery alternatives, focusing on symptoms of anxiety and depression and quality adjusted life years (QALYs). Generally, reimbursement models vary significantly depending on the country’s health system. While some countries, such as Norway, have a tax-funded, publicly administered reimbursement system, other countries have a fee-for-service system where providers are paid for each service delivered [23, 24]. Thus, in Norway, individuals first present to their general practitioner (GP) who act as a gatekeeper to the specialist services which may offer cognitive behavioral therapy. In addition, preventive programs may also be offered in schools where school health nurses provide the intervention. Reimbursement of healthcare interventions should be prioritized based on effectiveness (measured in QALYs), resource utilization (costs), and severity (measured by absolute shortfall, defined as the difference in accumulated QALYs in the current health condition relative to the general population) [25, 26]. We include the first two criteria in our cost-effectiveness analysis, where the results should be compared with a cost-effectiveness threshold (CET), reflecting the maximum threshold to pay per QALY gained. In Norway, the threshold varies with severity and ranges from NOK 275,000 to NOK 825,000 per QALY [27]. For symptoms of anxiety and depression, there is no such defined threshold.

We hypothesize that the least resource demanding alternatives, characterized by a hybrid delivery model (8 + 8 sessions), parental involvement with a psychoeducational brochure, and absence of feedback to group leaders, will be the most cost effective, maintaining comparable efficacy to more resource-intensive alternatives, as measured by symptomatology outcomes and QALYs.

## Method

The cost-effectiveness analysis of the eight strategies was conducted following Norwegian guidelines for priority settings as indicated in [25].

### Study design

The trial, on which we based the current study, examined three factors in a cluster-randomized 2 × 2 × 2 full factorial design, resulting in eight experimental conditions (see Table 1). The study aimed to optimize an intervention for the health services. We have detailed the methods employed in this study in the protocol paper [21] and effect publication [22], Therefore, the following method section provides a briefer overview, with references to these publications where applicable. Our design provided a unique opportunity to examine the cost of intervention delivery using real-world data that may inform decision-making, resource allocation and usability in the municipalities.

**Table 1:** The eight strategies

The eight strategies		Factor 1	Factor 2	Factor 3
		Child groups sessions Long/hybrid	Parental involvement High/low	Measurement feedback Feedback/no feedback
SLN	Short/low/no feedback	8 + 8	Parental brochure	No feedback
SLF	Short/Low/feedback	8 + 8	Parental brochure	Feedback
SHN	Short/high/no feedback	8 + 8	5 parental sessions	No feedback
SHF	Short/high/feedback	8 + 8	5 parental sessions	Feedback
LLN	Long/low/no feedback	16	Parental brochure	No feedback
LLF	Long/low/feedback	16	Parental brochure	Feedback
LHN	Long/high/no feedback	16	5 parental sessions	No feedback
LHF	Long/high/feedback	16	5 parental sessions	Feedback

### Intervention and the three components

Emotion is an indicated transdiagnostic group intervention based on CBT [2] for children aged 8–12 years, aimed at reducing symptoms of anxiety and depression by teaching the children coping strategies in groups with up to seven children. Group leaders were employed in municipal health services or worked with mental health in the school services. Following an initial effectiveness study with positive results [28], three promising factors were identified to optimize the intervention [29].

The first factor aimed to streamline the intervention by reducing the number of face-to-face sessions from 16 to 8, with the remaining sessions made digitally accessible online (hybrid version). Hence, some participants received a full 16 session version, and some received a hybrid version. The second factor involved modifying parental participation. Originally, parental involvement included five psycho-educational group sessions, conducted in parallel to the children’s 8-week course. Half of the groups received this version of parental involvement. The introduction of a self-help brochure based on the parental group meetings workbook, reduces the time spent by group leaders. We then implemented this delivery method in the remaining groups. The final factor was the implementation of a feedback system (MFS), allowing group leaders to track child progress and adapt the intervention as needed where half of the groups received this feedback system and half did not.

Table 1 displays the factors long delivery format/hybrid intervention, parental involvement (high/low) and Measurement Feedback System (MFS; Feedback/No Feedback) from the most to the least resource intensive strategies.

The three factors presented a total of 8 different strategies, Leading to a variability of working hours for the health services. This variability ranged from a total of 16 (SLN) hours for the strategy with the lowest labor intensity to 44.5 h for the highest (LHF), including additional time for supervision (4 h) and recruitment (2 h) per intervention group, see also Table 2.

**Table 2:** Resource utilization and costs according to strategy and cost components for the eight strategies

Experimental strategy	Child sessions^	Parent sessions^	Total hours, sessions + prep	MFS data review^	GL sessions w/ supervisor^	Recruitment and screening^	GL hourly cost NOK	Workbooks NOK	MFS app per group NOK	Supervision per group NOK	Cost per group w/ 2 GL NOK	Cost per child NOK ^^
SLN	8	0	16	0	4	2	529	1345	0	1000	25,621	5124.2
SLF	8	0	16	8	4	2	529	1345	225	1000	34,310	6862.0
SHN	8	5	28,5	0	4	2	529	2540	0	1000	40,041	8008.2
LLN	16	0	32	0	4	2	529	1345	0	1000	42,549	8509.8
SHF	8	5	28,5	8	4	2	529	2540	225	1000	48,730	9746.0
LLF	16	0	32	8	4	2	529	1345	225	1000	51,238	10247.6
LHN	16	5	44.5	0	4	2	529	2540	0	1000	56,969	11393.8
LHF	16	5	44.5	8	4	2	529	2540	225	1000	65,658	13131.6

*GL* Group Leader, ^hours spent, ^^ Costs reported in Norwegian krone, 100 NOK = 8.62 EUR (Dec. 2024) Parent sessions lasted 90 min, child sessions 60 min. Average number of children pr group = 5,* MFS* Measurement Feedback System

### Participants and recruitment

Each semester, the schools enrolled participants in up to five waves of recruitment, beginning spring 2020, following the recruitment procedure we described in the protocol [21].

The study involved the active participation of 52 public schools from both urban and rural areas, spanning 39 municipalities throughout Norway. The total sample size included children who agreed to participate in the study and who attended grades four through six (*N* = 701). Due to Covid 19, which coincided with the study’s start, we considered the first wave of children lost, and therefore conducted the analysis on children recruited in wave 2 to 5.

## Measures

### Health measures

#### The multidimensional anxiety scale for children (MASC-C)

The MASC-C [30] is a questionnaire designed to assess anxiety in children and adolescents between the ages of 8 and 19. It consists of 39 items measuring anxiety symptoms experienced during the past two weeks. Respondents rate each item on a scale from 0 to 3, (“0” for “never true about me,” “1” for “rarely true about me,” “2” for “sometimes true about me,” and “3” for “often true about me”). The total sum score on the MASC-C can range from 0 to 117, with higher scores indicating higher levels of anxiety.

The MASC-C has demonstrated good psychometric properties, including high retest reliability [31], as well as good to excellent interrater reliability in Norwegian clinical samples [32]. The questionnaire has also shown predictive and discriminative validity in various studies [33–35]. Elevated scores on the MASC-C have been significantly associated with meeting diagnostic criteria for anxiety disorders in a Norwegian sample [32], and the factor structure has been replicated in a Norwegian sample of school children [36]. In the current study, Cronbach’s alpha was 0.86.

#### The short mood and feelings questionnaire (SMFQ-C)

The SMFQ-C [37] assesses depressive symptoms in children and adolescents between the ages of 8 and 18. It consists of 13 questions measuring cognitive, affective, and behavioral-related symptoms of depression experienced during the past two weeks. Respondents rate each statement as ‘true’ (scored as 2), sometimes true’ (scored as 1), or ‘not true (scored as 0), yielding a sum score ranging from 0 to 26.

The longer version of the MFQ has been validated in both clinical and general population samples [38, 39], and the SMFQ has also been validated in diverse populations [37, 40]. In the present study, Cronbach’s alpha was 0.84.

#### Health related quality of life (HRQoL)

We used the ten items in Kidscreen-27 [41], corresponding to the dimensions included in the Kidscreen-10 Index to transform Kidscreen-10 into a measure of Health related Quality of life (HRQoL) using the algorithm developed by Chen, Stevens [42]. The Kidscreen is a measure of quality of life for youth aged 8 to 18 years that has been validated in several European countries. The ten items from Kidscreen-27 that comprise Kidscreen-10 are item), item 2 (*fit and well*), item 5 (*energy*), item 9 (*sad*), item 11 (*lonely*), item 13 (*had enough time for yourself*), item 14 (*been able to do things that you want to do in your free time*), item 16 (*parent(s) treated you fairly*), item 21 (*had fun with friends*), item 25 (*got on well at school*) and item 26 (*been able to pay attention*). The items have five levels from “*not at all”* to “*a large extent.”* Kidscreen-10 has been validated in several European countries [41]. Cronbach’s alpha for Kidscreen-10 was 0.76.

### Costs

The health care perspective determined which costs were included in the cost-estimations and included costs of delivering the intervention. To estimate the cost per child, our calculations assumed that each intervention group would include 5 children, as this was the average number observed in the main Echo trial [22]. We first considered the number of child-group sessions and the number of parent sessions within each condition. Based on the experience from the previous studies, we also made the assumptions that: (1) there would be/were two group leaders per group, (2) group Leaders in MFS conditions would dedicate one hour per week during the 8-week intervention period to review MFS data, (3) each group Leader would receive 4 h of supervision during the intervention period sharing the cost of supervision with other group leaders being supervised, (4) group Leaders would spend 2 h each on recruitment for groups and symptom screening, including communication with parents regarding their child´s participation in a group, (5) group leaders spent an hour of preparation per session. The cost per hour for a group leader varied according to profession and salary. In the estimation we used the average hourly wage of school health nurses, which was the main professional category in the current study (for current salary rates: https://www.nsf.no/lonn-og-tariff/statistikk).

The hourly wage was multiplied by a constant of 1.4 to account for social costs. In addition, cost associated with the MFS app, and child and parent workbooks were included.

The alternatives differed in the total number of hours spent by the group leaders running the intervention, hence the long version, with high parental involvement and feedback provided was the most resource intensive condition incurring the highest costs. The composition of cost per child is reported in Table 2.

### Statistical analysis

#### Health outcomes

As Kidscreen is a non-preference-based measure of HRQoL, QALYs were estimated by combining HRQoL and time. MASC, SMFQ, and QALYs were reported as accumulated means over the observation period. The collection of data for MASC, SMFQ and Kidscreen-10 was conducted at T1 (inclusion), T2 (12 weeks) and T3 (50 weeks), in total 62 weeks.

Correlations between the primary outcome measures (MASC, SMFQ and HRQoL) at T1 were calculated using Pearson’s correlation coefficient.

Mean and accumulated scores over time of the main outcome variables (MASC, SMFQ and HRQoL) were calculated at T1, T2, and T3.

Based on these observation points, for each of the measures the area under the curve was estimated. As an example, see the formula for calculation of accumulated group means for QALYs for the 62-week period as the period from T1 – T2 was 12 weeks and the period from T2 to T3 was 50 weeks [43].

$$\begin{aligned} & {\text{Accumulated}}~{\text{means}}~{\text{QALYs}}~ \\ & {\text{ = }}\frac{{{\text{HRQoL}}\left( {T1} \right) + {\text{HRQoL}}\left( {T2} \right)}}{2}*\frac{{12}}{{52}} \\ & + \frac{{{\text{HRQoL}}\left( {T2} \right) + {\text{HRQoL}}\left( {T3} \right)}}{2}*\frac{{50}}{{52}} \\ \end{aligned}$$

Incremental Cost Effectiveness ratio (ICER):

We conducted three separate analyses according to health outcome (MASC, SMFQ, QALYs). The eight strategies were ordered according to costs (from lowest to highest) with the related health outcome. Then, the ICER for health outcome (o), for the comparison of strategy *j + 1* versus *j* is defined by:

$$\begin{gathered} {\text{ICER}}_{{\text{o}}} \hfill \\ = \frac{{{\text{Cost}}_{{j + 1}} - {\text{Cost}}_{j} }}{{{\text{Health~Outcome}}_{{j + 1}}^{o} - {\text{Health~Outcome}}_{j}^{o} }} \hfill \\ = \frac{{\Delta {\text{C}}_{{j + 1,j}} }}{{\Delta {\text{E}}_{{j + 1,j}}^{o} }} \hfill \\ \end{gathered}$$

where superscript *o* is MASC, SMFQ-C and QALYs, *j* is the strategies 1 to 7,

and $\:\varDelta\:{\text{E}}_{j+1,j}^{o},$ are the incremental costs and the incremental effects for outcome o and comparison *j and j+1*. When the health outcome is measured with MASC and SMFQ, the interpretation of the ICER will be increased cost per unit of improvement (reduction) in MASC/SMFQ, and for QALYs, increased cost per QALY gained.

The estimated ICERs will be presented by the cost-effectiveness frontier, a graphical representation assessing the value of healthcare interventions by comparing costs and effects [44]. This enables a definition of the frontier consisting of the most efficient strategies. To identify the frontier, all strategies are organized from lowest to highest costs. The strategies that do not define the frontier either have higher costs *and* lower health outcomes (dominated strategies), or that alternative strategies provide a lower ICER. The term ‘extended dominated, ED’ is used when a strategy is both more costly and more effective, but of less value than the alternative strategies. For the analysis with QALYs, the ICER is compared with the Norwegian cost-effectiveness threshold (CET) as described earlier in the paper.

Statistical analysis was conducted using R version 4.4.1.

## Results

A total of 701 children participated in the study, with 419 (59.8%) being girls. The average age of the participants was 10.5 years, and they were recruited from fourth to sixth grade.

### Health outcomes

Table 3 reports the correlations between the primary health measures at T1.

**Table 3:** Correlation of primary health measures at T1

		MASC T1	SMFQ T1
MASC T1	Pearson correlation		
	N	701	
SMFQ T1	Pearson correlation	0.438**	
	p-value	< 0.001	
	N	701	701
HRQoL	Pearson correlation	− 0.368**	− 0.597**
	p-value	< 0.001	< 0.001
	N	695	695

*MASC* Multidimensional Anxiety scale – child version (March, 1997), *SMFQ* Short Mood and Feelings questionnaire – child version [37], *HRQoL* Health Related quality adjusted life years

At T1, there was a significant positive correlation between MASC and SMFQ, and significant negative correlations between these and HRQoL. Table 4 reports the means and estimated outcomes over the study period according to health outcomes and eight strategies .

**Table 4:** Mean and accumulated score over time of main outcome variables

Strategies		T1 MASC	T2 MASC	T3 MASC	Accumulated score over time MASC	T1 SMFQ	T2 SMFQ	T3 SMFQ	Accumulated score over time SMFQ	T1 HRQoL	T2 HRQoL	T3 HRQoL	Accumulated score over time HRQoL
SLN	N	109	98	83		109	98	83		108	98	82	
	Mean	73.25	61.78	57.61		11.72	10.24	9.11		0.75	0.79	0.79	
	SD	14.03	18.11	19.13	72.98 ^	5.75	7.10	6.41	11.84	0.10	0.12	0.13	0.94
SLF	N	102	95	77		102	94	77		101	94	77	
	Mean	70.46	56.31	52.19		11.99	9.38	8.88		0.75	0.81	0.79	
	SD	15.87	16.50	18.82	66.79	6.25	6.13	5.91	11.25	0.12	0.11	0.11	0.95
SHN	N	61	56	50		61	56	50		60	56	50	
	Mean	70.77	60.98	57.96		11.49	8.43	7.86		0.74	0.81	0.81	
	SD	12.88	16.76	19.31	72.39	5.93	5.72	5.42	10.13	0.11	0.12	0.12	0.96
LLN	N	84	70	68		84	70	68		84	68	68	
	Mean	65.37	57.54	52.09		11.21	7.83	7.88		0.75	0.82	0.82	
	SD	14.05	16.97	16.75	66.89	4.58	5.75	5.79	9.75	0.10	0.11	0.11	0.97
SHF	N	57	52	48		57	52	48		56	50	48	
	Mean	71.32	60.5	52.73		11.56	9.42	8.00		0.74	0.78	0.8	
	SD	15.62	19.33	22.03	69.65	5.44	5.88	6.72	10.8	0.09	0.12	0.11	0.94
LLF	N	104	99	84		104	99	82		103	99	82	
	Mean	67.21	55.69	53.56		11.26	8.76	7.68		0.76	0.81	0.82	
	SD	14.29	17.17	18.55	66.70	4.99	5.94	6.12	10.21	0.10	0.12	0.12	0.97
LHN	N	88	80	78		88	80	78		88	80	78	
	Mean	68.94	59.76	55.09		11.18	8.60	7.65		0.75	0.81	0.83	
	SD	15.37	15.18	17.05	70.07	5.04	5.49	5.50	10.1	0.10	0.12	0.12	0.97
LHF	N	96	83	76		96	83	75		95	83	73	
	Mean	69.36	55.67	54.13		11.75	8.88	7.96		0.75	0.81	0.81	
	SD	14.98	17.42	17.04	67.22	5.42	5.83	5.25	10.48	0.10	0.11	0.11	0.95
Total	N	701	633	564		701	632	561		695	628	558	
	Mean	69.56	58.30	54.40		11.53	9.00	8.16		0.75	0.81	0.81	
	SD	14.81	17.22	18.47		5.43	6.06	5.89		0.11	0.11	0.12	

*MASC* Multidimensional Anxiety scale – child version (March, 1997), *SMFQ* Short Mood and Feelings questionnaire – child version [37], *HRQoL* Health Related quality adjusted life years.

General trends showed that both MASC and SMFQ scores decreased from T1 to T3 across all strategies, indicating symptom reduction. HRQoL scores remained relatively stable over time, but we noted small positive changes in participants’ quality of life over time.

### Cost-effectiveness analysis

**Fig. 1:**
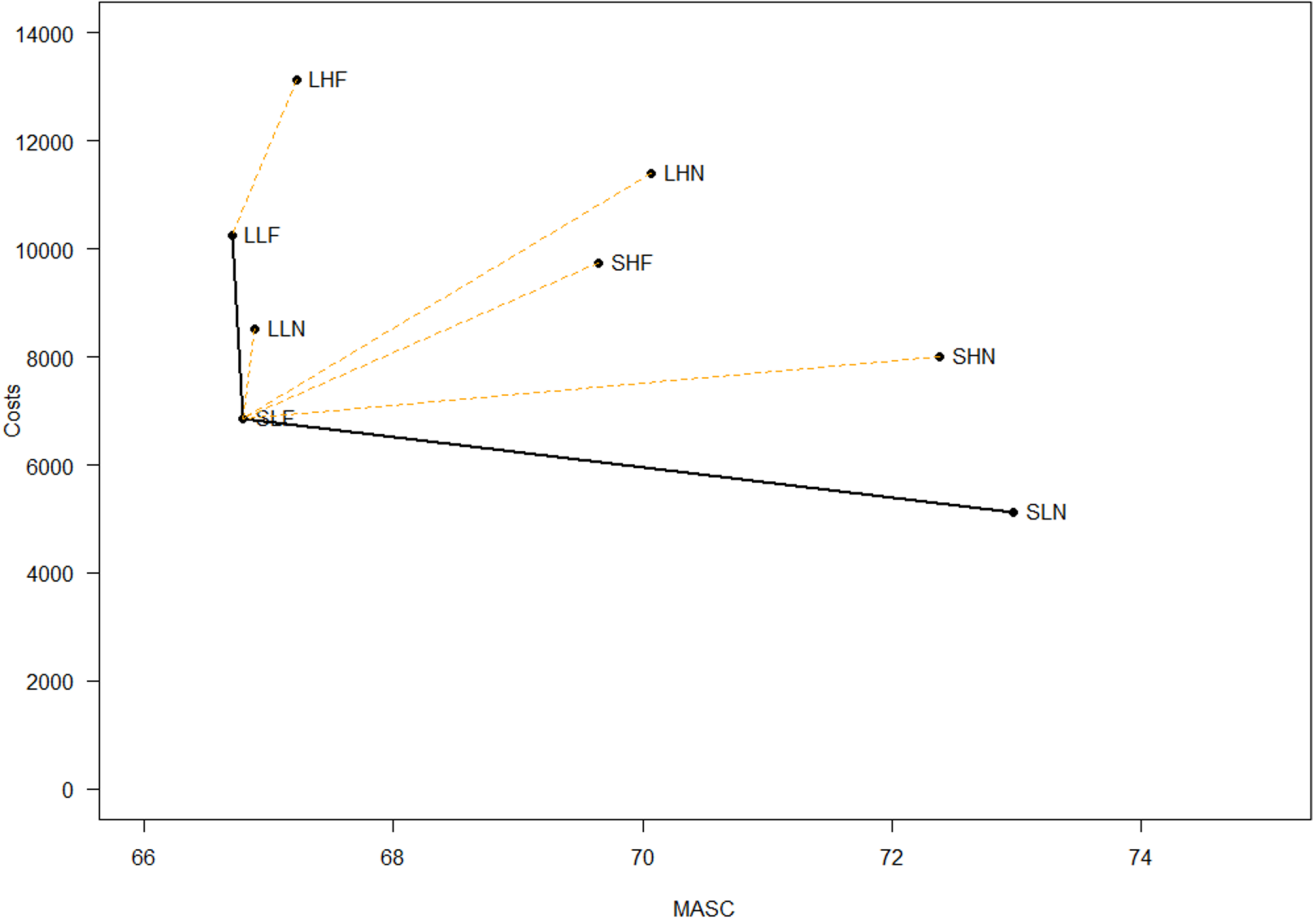
The cost-effectiveness frontier for MASC outcome. *MASC* Multidimensional Anxiety scale – child version (March, 1997), *SLN* Hybrid version/low parental involvement/No feedback, *SLF* Hybrid version/low parental involvement/feedback, *LLF* Long version/Low parental involvement/feedback

**Fig. 2:**
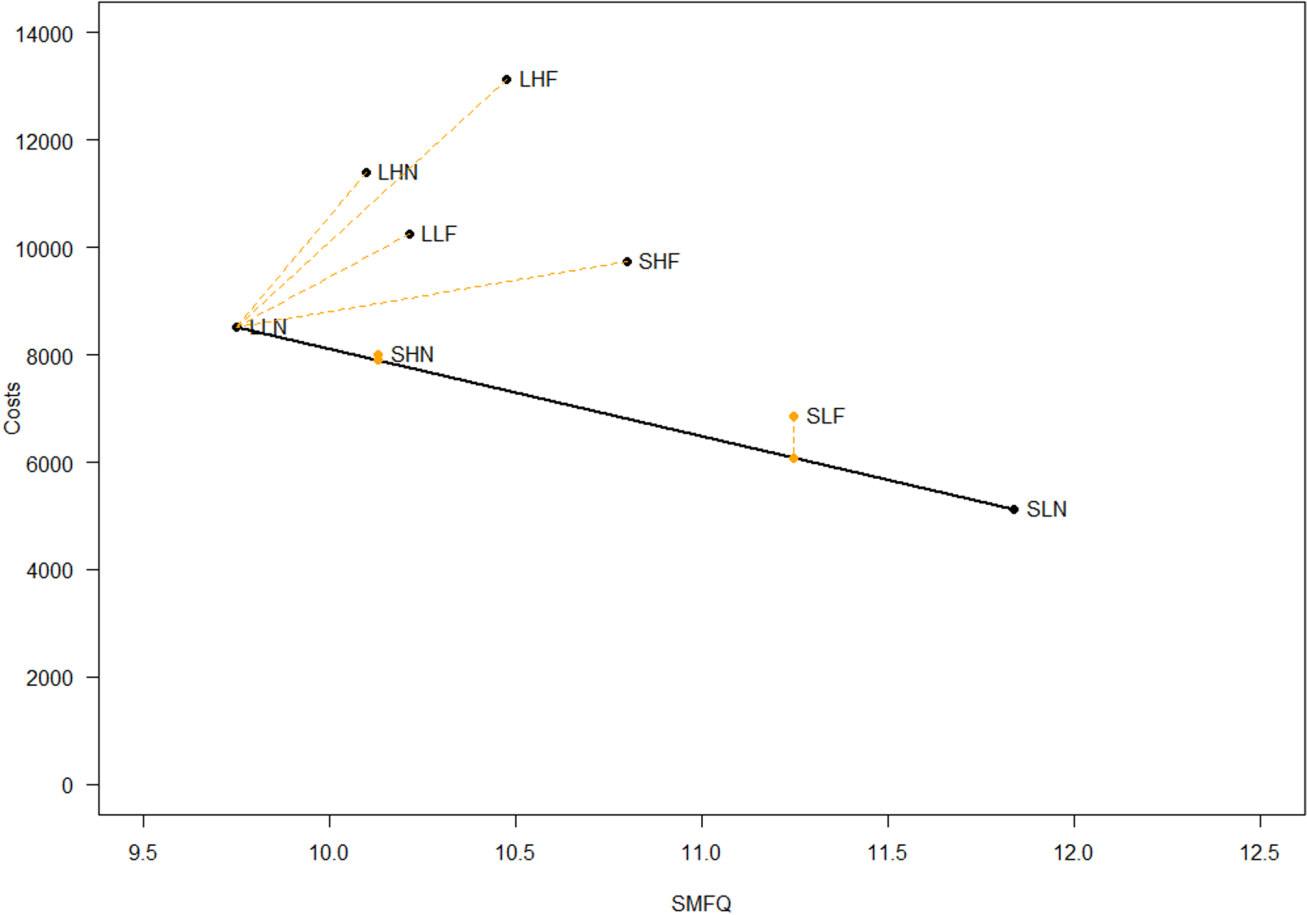
The cost effectiveness frontier for SMFQ. *SMFQ* Short Mood and Feelings questionnaire – child version [37], *SLN* Hybrid version/low parental involvement, No feedback, *SHN* Hybrid version/high parental involvement/feedback, *LLN* Long version/Low parental involvement/No feedback

**Fig. 3:**
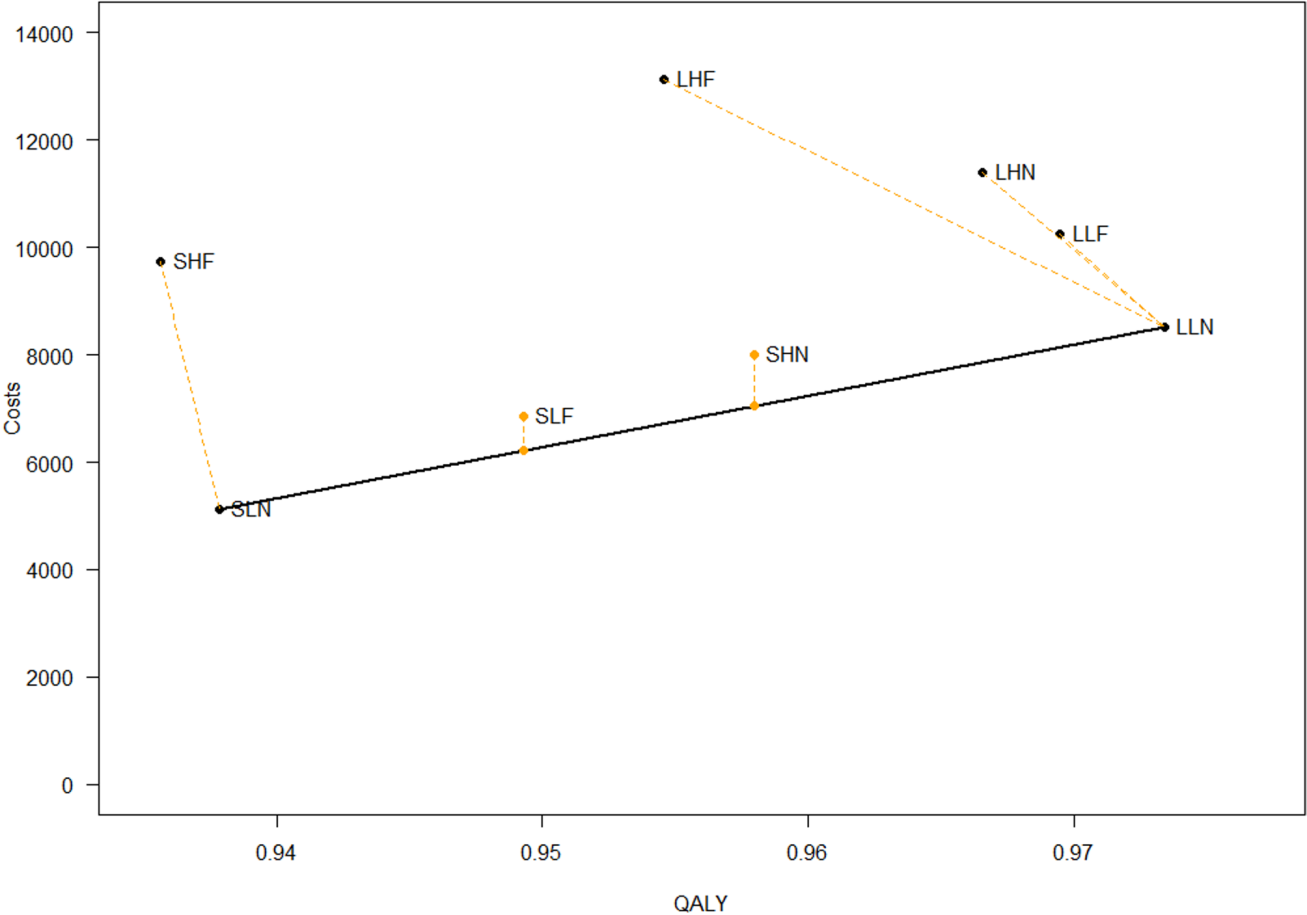
The cost effectiveness frontier for QALYs. *QALY* Quality adjusted life years, *SLN* Hybrid version/low parental involvement, No feedback, *SLF* Hybrid version/low parental involvement/feedback, *SHN* Hybrid version/high parental involvement/feedback, *LLN* Long version/Low parental involvement/No feedback

We report the ICER and cost-effectiveness frontier according to strategy and health outcome in Tables 5, 6 and 7; Figs. 1, 2 and 3, for MASC, SMFQ and QALYs, respectively.

**Table 5:** Cost-effectiveness results for MASC, costs per child and ICER in NOK

Strategy	Costs	Incr. Costs	Accum. MASC	Incr. MASC	ICER	Incr. Cost Frontier	Incr. MASC Frontier	ICER Frontier
SLN	5124		72.98					
SLF	6862	1738	66.79	6.19	281	1738	6.19	281
SHN	8008	1146	72.39	− 5.6	− 05			D
LLN	8510	502	66.89	5.5	91			ED
SHF	9746	1236	69.65	− 2.76	− 448			D
LLF	10,248	502	66.70	2.94	170	3386	0.09	38,712^
LHN	11,394	1146	70.07	− 3.37	− 341			D
LHF	13,132	1738	67.22	2.85	610			D

*Incr.* Incremental,* Acc.* Accumulated,* ED* Extended dominated strategy,* D* Dominated strategy, ^the ICER is -(10248 − 6862)/(66.70-66.79) = 37.622 (The deviation from the correct ICER,38712, is due to rounding)

**Table 6:** Cost-effectiveness results for SMFQ, costs per child and ICER in NOK

Strategy	Costs	Incr, Costs	Acc. SMFQ	Incr. SMFQ	ICER	Incr. Cost Frontier	Incr. SMFQ Frontier	ICER Frontier
SLN	5124		11.84					
SLF	6862	1738	11.25	0.59	2938			ED
SHN	8008	1146	10.13	1.12	1025			D
LLN	8510	502	9.75	0.38	1324	3386	2.09	1621^
SHF	9746	1236	10.80	-1.05	− 1180			D
LLF	10,248	502	10.21	0.58	859			D
LHN	11,394	1146	10.10	0.12	9805			D
LHF	13,132	1738	10.48	-0.38	-4581			D

* Incr.* Incremental,* Acc.* Accumulated,* ED* Extended dominated,* D* Dominated strategy, ^ the ICER is -(8510 − 5124)/(9.75–11.84) = 1620 (The deviation from the correct ICER, 1621, is due to rounding)

**Table 7:** Cost-effectiveness results for qalys, costs per child and ICER in NOK

Strategy	Costs	Incr. Cost	Acc. QALY	Incr. QALY		ICER		Incr. Cost Frontier		Inc. QALY Frontier		ICER Frontier	
SLN	5124		0.938										
SLF	6862	1738	0.949	0.011		151,740						ED	
SHN	8008	1146	0.958	0.009		132,112						ED	
LLN	8510	502	0.973	0.015		32,489		3386		0.036		95,188^	
SHF	9746	1236	0.936	-0.038		- 32,751						D	
LLF	10,248	502	0.969	0.034		14,820						D	
LHN	11,394	1146	0.967	-0.003		− 392,548						D	
LHF	13,132	1738	0.955	-0.012		− 145,065						D	

*Incr.* Incremental, *Acc.* Accumulated, *ED* Extended dominated strategy, *D* Dominated strategy, ^ the ICER is -(8510 − 5124)/(0.973 − 0.938) = 94,055 (The deviation from the correct ICER, 95188, is due to rounding)

### MASC- anxiety symptoms

Table 5 presents the eight strategies given the costs per child and outcomes of the eight different alternatives.

In Table 5 we identify SLN, SLF and LLF as the three efficient strategies defining the frontier, with the Short/hybrid solution with low parental involvement and no feedback (SLN) being the least resource intensive (NOK 5124), but also having one of the lowest MASC outcomes (73.0). When comparing with SLN, SLF (short/hybrid, low parental involvement, and feedback) has an incremental cost of NOK 1738, and an incremental reduction in MASC of 6.19, providing an ICER of NOK 281 per MASC point improvement. When compared with SLF, LLF has an incremental cost of NOK 3386 and an incremental reduction in MASC of 0.09, providing an ICER of NOK 38,712 per MASC point improvement.

Figure 1 depicts all experimental conditions in the cost-effectiveness frontier (Fig. 1), allowing for visual inspection of all alternatives: the conditions SLN, SLF and LLF are on the frontier, while all other alternatives are either extended dominated (LLN) or dominated compared to the preferred alternatives.

### SMFQ

Table 6 presents the costs, outcomes and ICER for SMFQ.

Table 6 shows that SLN and LLN define the frontier when the health outcome is SMFQ. Compared with SLN, LLN has an incremental cost of NOK 3386, with an incremental reduction in SMFQ of 2.09, providing an ICER of NOK 1621 per SMFQ point improvement (higher costs and improved SMFQ score).

The least resource demanding alternative (SLN) remains an efficient alternative as with MASC. However, with SMFQ, the long group version with low parental involvement (LLN), emerged as an efficient alternative, while the other conditions are dominated. Among these, we found that SLF and SHN are extended dominated, with SHN positioned only slightly above the frontier.

### QALYs

Table 7 presents costs, QALYs and ICER for all strategies.

SLN and LLN define the frontier. The other strategies are either dominated or extended dominated and are thus not considered cost-effective. Compared with SLN, LLN has a higher incremental cost of NOK 3386, and increased QALY of 0.036, providing an ICER for LLN compared to SLN of NOK 95,188 per QALY gained, which is below the lowest CET value (NOK 275,000).

Examining Fig. 3, we can visually see thathe least resource demanding alternative (SLN) holds as an efficient alternative, similar to what we found with anxious symptoms (MASC) and depressive symptoms (SMFQ). The LLN (long, low parental involvement and feedback alternative) strategy also appears on the frontier when examining depressive symptoms (SMFQ).

Based on the results of the three analyses, we identify the strategies positioned on the frontier for different health outcomes: LLF, SLF, and SLN for anxiety as measured by the MASC; LLN and SLN for depression as measured by the SMFQ and for quality-adjusted life years (QALYs).

## Discussion

This paper examined the cost-effectiveness of eight program delivery strategies, designed to alleviate symptoms of anxiety and depression among young children taking a health care perspective. As highlighted in previous research [9], the scarcity of resources in youth mental health necessitates budget al.location based on cost-effectiveness analyses to maximize health outcomes in the population. It is important to find a balance between the allocation of sufficient resources to reduce symptoms and prevent the onset of mental disorders, without allocating unnecessary resources that alternatively could have been used elsewhere.

Our analysis revealed that LLN, the long version with low parental involvement and no feedback, and SLN (the hybrid version, with low parental involvement and no feedback) emerged as efficient strategies for reducing depressive symptoms and improving QALY. While not on the frontier, SHN, the hybrid version with parental involvement and no feedback, was very close to being cost effective and could, in some instances, also be a possible strategy. For anxiety symptoms, slightly altered preferences were discerned, favoring the LLF (long version with low parental involvement and feedback) and the SLF (the hybrid version with low parental involvement and feedback) in addition to SLN as these strategies were on the cost-effectiveness frontier. Our hypothesis that the least resource-demanding alternatives would be the most cost effective was partially supported; however, the more resource-demanding alternatives also proved to be cost-effective.

Examining the results more closely for anxiousness, the cost-effectiveness frontier reveals that SLN, SLF, and LLF are all on the frontier. This means these interventions are among the cost-effective options available. Comparing the different results, however, reveal that it appears to be important to determine resource intensity versus effectiveness as the SLN (short/hybrid solution with low parental involvement and no feedback) is the least resource-intensive intervention. However, it also results in the least reduction in anxiety symptoms (6.2 points Less compared to SLF and 6.3 points less compared to LLF). This indicates a trade-off between cost and effectiveness regarding symptom reduction. The SLF (short/hybrid, low parental involvement, and feedback) has higher costs than the SLN alternative, but results in greater symptom reduction. Of these two cost effective alternatives, SLF therefore emerges as the preferred intervention as it is a better balance between costs and reductions in symptoms. This suggests that the addition of feedback, even with slightly increased costs, improves the intervention’s effectiveness. Comparing the two other cost-effective alternatives, SLF and LLF, although LLF has higher costs than SLF, it offers only marginally better reductions in anxiety symptoms (difference of 0.09). This implies that the additional investment in LLF does not yield substantially better outcomes compared to SLF, reinforcing that SLF is the preferred cost-effective alternative. These results align with Lee, Barendregt and collegues [14], who found that school-based, internet delivered preventive interventions can be more cost effective compared to the more resource-demanding face-to-face interventions. Examining accumulated means over an extended period as is done in the current analysis makes it possible to capture sustained changes in outcomes measured by the MASC. Establishing the clinical significance of these changes remains a challenge, but is important in a preventive context where even small reductions in symptoms can be meaningful and especially so if they result in improved quality of life and better functioning. However, there are no standard methods for determining what should be used as a cutoff for clinical relevance. A difference of 6.2 points between two alternatives that both are on the cost effectiveness frontier may be clinically meaningful in this preventive setting. This perspective is reinforced by the overall reduction observed in MASC scores from T1 to T2 in this study as reported in Lisøy, Neumer [22], where reductions varied between 10.09 and 12.60 points. The MASC has demonstrated good psychometric properties [31]. Studies also show that even moderate score changes may be associated with improvements in functioning, relationships, and school performance [45]. Hence a 6.2 point reduction between cost effective alternatives could indicate a meaningful change with improved management of the youth’s health.

When evaluating options near the frontier, the strategy LLN (long with low parental involvement and no feedback) is “Extended dominated”. This indicates that, although LLN is not the least effective option when compared to the alternatives, it falls short in cost-effectiveness relative to the strategies on the frontier. In other words, LLN is not a preferable choice as there are other strategies that provide equivalent or better outcomes at lower or comparable costs. We therefore exclude LLN from consideration when optimizing resource allocation in the services.

Examining the cost effectiveness results for depressive symptoms, the SLN and LLN were positioned on the cost-effectiveness frontier (Fig. 2), indicating they are the most efficient use of resources given their cost and effectiveness profiles. Upon closer analysis, the intervention SLN (Hybrid version with low parental involvement and no feedback) is the least costly option. However, in the same way as for anxiousness, it also provides the lowest change in accumulated symptoms of depression compared to LLN. This means that while SLN might be preferred in scenarios where resources are particularly scarce, its effectiveness in reducing symptoms is smaller. The intervention LLN (Long version with low parental involvement and no feedback) has the highest costs among the two preferred interventions, but also offers the greatest reduction in symptoms, with a difference of 2.1 points on the SMFQ compared to the SLN. This makes LLN the best option for maximizing symptom reduction, but at a higher cost. While the strategy SHN (Hybrid version with high parental involvement and feedback) is not on the frontier, it is worth mentioning as a possible alternative. This strategy has higher costs than SLN, but it provides an additional symptom reduction of 1.7 points on the SMFQ, and the additional symptom reduction compared to LLN is only 0.4 points. This suggests that SHN might be a strategy to consider due to its greater efficacy in reducing symptoms if additional resources are available although it is just extended dominated. Again, it is not well established what a meaningful difference for depressive symptoms could be in this preventive context. The SMFQ has been validated in diverse populations and demonstrates strong psychometric properties [34]. Furthermore, studies indicate that the SMFQ is sensitive to change: for instance, Thabrew and colleagues [46] reported that the SMFQ exhibits ‘satisfactory sensitivity to change in a help-seeking adolescent sample with mild to moderate symptoms”. Examining the changes in scores for all the different delivery strategies as reported in Lisøy, Neumer [22], the reductions from T1 to T2 ranged between 2.36 to 2.70, and a difference in symptoms reductions in the cost-effective alternatives on the frontier of up to 2.09 points could most likely be clinically important as they are close to the actual reductions achieved from pre to post intervention. All other interventions are dominated by SLN and LLN, meaning they provide less cost-effective solutions.

The cost-effectiveness analysis for quality adjusted life years (QALYs) reveals that, similar to the findings for symptoms of anxiety (MASC) and depression (SMFQ), the intervention SLN (Hybrid version with low parental involvement and no feedback) is on the cost-effective frontier (Fig. 3). SLN incurs lower costs compared to alternative strategies, although it offers lower QALYs than the other options. This suggests that while SLN is affordable, its effectiveness in improving QALY is lower. The intervention LLN (Long version with low parental involvement and no feedback) is also considered cost-effective. It provides a higher QALY at a reasonable incremental cost (NOK 3386), positioning it as a favorable alternative for enhancing quality of life while maintaining cost efficiency. Other strategies such as SHF, LLF, LHF, and SLF are not on the cost-effectiveness frontier. SLF and SHN are however categorized as extended dominated, and remain close to the frontier, suggesting a degree of cost effectiveness under certain conditions. Both strategies align with the results of the SMFQ and the MASC, indicating their proximity to the frontier and potential as alternative options.

Examining the results in relation to the Norwegian threshold per QALY set to NOK 275,000 [25], the cost for the most expensive strategy is 95,188, which is well within the acceptable range.

The SLN remains a preferred option due to its low resource demands, and aligns with our findings for (MASC) and (SMFQ). The LLN intervention also holds a position on the frontier when examining depressive symptoms, reaffirming its cost-effectiveness in various outcome measures. Another short-duration strategy, SHN, involving parents with five parental meetings, lies close to the frontier, reinforcing its alignment with SMFQ outcomes. SLF, though extended dominated, aligns comparably with the cost effectiveness results of MASC.

Healthcare decision-makers should consider that while SLN has the lowest costs, LLN is also on the frontier and offers a better quality of life. Emphasizing these interventions can yield notable improvements in health outcomes, justifying their costs when resources permit.

In the analysis we included costs related to the intervention and its delivery, hence other costs related to the use of healthcare services and/or social services are not included. This leads to an underestimation of the costs for each child in the trial. Whether inclusion of these costs would result in changes in incremental costs is uncertain. If the use of services declines with improved health, incremental costs are overestimated. On the other hand, increased knowledge and follow-up can also initiate increased demand for services and help, which could increase the incremental costs.

We should also bear in mind that for youths in particular, the economic impact of poor mental health extends beyond healthcare costs to sectors such as education and justice [47–49]. The lack of investments in prevention, as pointed out by McDaid, Park [50] may be due to exactly the fact that prevention often involves multiple sectors where the costs may be incurred in one sector, while the benefits are incurred in another sector, and often later, or that the sector does not have improving mental health as its primary objective. For instance, schools prioritize education and may lack resources for preventive interventions, and the health sector benefits do not directly impact the educational sector.

Examining the association of the variables, the moderate positive correlation at T1 between MASC and SMFQ was 0.438, *p* < 0.001, suggesting that higher anxiety symptoms tend to be associated with higher depressive symptoms. We found a correlation coefficient between MASC and HRQoL of -0.368, indicating that higher anxiety symptoms tend to be associated with lower HRQoL. Lastly, the correlation coefficient between SMFQ T1 and QALYS T1 was − 0.597, which indicates a strong negative correlation, suggesting that higher depressive symptoms tend to be associated with lower HRQoL. The significant correlations between these primary outcomes indicate the interconnected nature of mental health symptoms and overall quality of life. Interventions targeting reductions in anxiety and depressive symptoms could potentially lead to improvements in quality of life.

The comprehensive cost-effectiveness analysis across different measures—anxiety symptoms (MASC), depressive symptoms (SMFQ), and QALYs, provides guidance on how to optimize the allocation of resources for mental health interventions.

Given intermediate resources, interventions like SLF provide a balance between cost and effectiveness and should be prioritized over less effective yet cheaper strategies like SLN. A focus on SLF can lead to better health outcomes while ensuring that the additional resources invested lead to important improvements in symptom reduction for anxiety. Furthermore, SHN also strikes a balance between cost and reduction of symptoms and may be a possible alternative. This aligns with principles of health economics and effective resource utilization.

For resource-limited settings SLN may serve as a choice due to its low cost, despite its modest effectiveness. This may for instance be the case with limited personnel in primary health services. To provide a more cost-effective intervention where reach is improved and with acceptable reductions in symptoms is obtained, may therefore ensure that research-based interventions are adopted in the health services.

LLN, however, is preferred in more ideal situations where the priority is health outcomes over costs, justifying its superior reduction in depression symptoms.

Strengths and Limitations.

The trial’s large sample size with participants from both urban and rural schools across the country, enhances the generalizability of the findings. The innovative factorial design allowed for a comparison of different delivery strategies of an intervention targeting common mental health problems in youth. Child anxiety and depression symptoms were assessed using well established and validated measures. Additionally, the study achieved satisfactory child response rates across all three timepoints, ensuring reliable longitudinal data. The trial, conducted within a real-world school setting and utilizing first-line school health nurses as group leaders, supports the practical interpretation of results and the potential for translating research into practice.

However, the study also presents limitations:

While researchers have recommended factorial designs in new and innovative optimization frameworks, few cost-effectiveness studies have employed this design. As a result, there are no well-established guidelines for examining cost-effectiveness specifically tailored to such designs.

The absence of a control group in this study receiving no strategy or combination of strategies, which could be compared to the eight different intervention delivery strategies, is another notable limitation. Control groups are commonly used to establish baseline measures in cost-effectiveness analysis, offering a point of comparison for evaluating incremental benefits of interventions, while in this study we compared cost effectiveness of eight different alternatives.

Furthermore, examining costs and outcomes using the Incremental Cost-Effectiveness Ratio (ICER) may pose challenges. Although researchers can adapt ICER to a factorial design, they typically use it to compare an active intervention against a control condition. For factorial studies involving multiple strategies, there is no established consensus on the best method to determine cost-effectiveness [20]. According to Dziak [20], the fundamental concepts of the cost-effectiveness frontier still apply, but it remains a dilemma between developing a complex versus a parsimonious model, each having its own set of advantages and disadvantages.

Another important consideration is the choice of comparator strategies included in the study. As pointed out by O’Mahony, Naber [51], the ICER will depend both on costs and effects of the strategy, as well as on which strategies researchers include for comparison. While we included strategies based on a rationale of examining different strategies for ensuring effectiveness and delivery of the intervention, we cannot rule out that other strategies could have been relevant to include. While cost-effectiveness from a health service perspective was the aim of this study, the results could possibly have presented otherwise if all costs, including costs of parental involvement and parental absenteeism from work related to the child’s problem, use of other healthcare and social services, had been included.

In the analyses above, results are based on mean values, not taking uncertainty in the health outcomes and costs into account. Uncertainty in the health outcomes could have been explored by the variation in individual health outcome scores according to strategies. However, regarding costs this was not possible as we estimated costs per strategy, not varying between individuals. With more detailed information on costs, additional analyses would be relevant to include.

Lastly, although validated measures for clinical outcomes were used, we derived the calculation of Quality-Adjusted Life years (QALYs) from ten items of the Health-Related Quality of Life (HRQoL) measure Kidscreen, using an algorithm developed by Chen et al., 2014 for this purpose. This reliance on a subset of items, and a specific algorithm may introduce some degree of uncertainty in the assessment of QALYs. The use of QALYs is the recommended outcome measure according to Norwegian guidelines for priority setting, but where EQ-5D-5 L is the preferred instrument [52]. Whether EQ-5D-5 L would derive at similar findings would have to be analyzed in future studies.

## Conclusion

To conclude, we recommend the least resource-intensive combination of strategies, SLN (Short/Low/No Feedback), for highly resource constrained environments, ensuring limited yet cost-effective symptom reduction. The long version, with low parental involvement and no feedback, LLN, should be chosen when symptom reduction is a high priority and cost is less of a concern. Delivery methods such as SHN (Short/High/No Feedback) and LLN can lead to substantial clinical improvements and justify their higher costs when resources permit, optimizing health outcomes in accordance with cost-effectiveness principles.

In preventive care settings, however, decisions related to the importance of reach and resources available also need to be taken into consideration. Healthcare decision-makers should consider the specific resource availability and clinical goals to determine the most appropriate interventions, emphasizing those strategies providing substantial health benefits for the investment made.

## Data Availability

Data will be made available upon reasonable request once the project is completed and the data is fully anonymized. The syntax for the statistical analysis can be accessed freely on https://github.com/ToreWentzel-Larsen/ECHO-cost-effectiveness/.

## Abbreviations

CBT: Cognitive behavioral therapy
HRQoL: Health related quality of life
ICER: Incremental Cost Effectiveness ratio
LLN: Long delivery format/low parental involvement/no feedback
LLF: Long delivery format/low parental involvement/feedback
LHN: Long delivery format/high parental involvement /no feedback
NOK: Norwegian Krone), approx. 1 USD = 10.2 NOK, 1 EUR = 11.2 NOK (as of August 2025)
SLN: Short delivery format/low parental involvement/no feedback
SLF: Short delivery format/low parental involvement/no feedback
SHN: Short delivery format/high parental involvement /no feedback
SHF: Short delivery format/high parental involvement /feedback
MASC-C: The Multidimensional Anxiety Scale for Children
MOST: The Multiphase Optimization Strategy
SMFQ: The Short Mood and Feelings Questionnaire
QALY: Quality-Adjusted Life-Years
